# Perceived training gaps of newly licensed veterinarians and impact of participation in a peer-facilitated quality assurance program in Ontario, Canada: interviews with peer advisors

**DOI:** 10.3389/fvets.2025.1725153

**Published:** 2026-01-09

**Authors:** Michael W. Brunt, Kate Wycherley, Basem Gohar

**Affiliations:** 1 Department of Population Medicine, Ontario Veterinary College, University of Guelph, Guelph, ON, Canada; 2 Department of Clinical Studies, Ontario Veterinary College, University of Guelph, Guelph, ON, Canada; 3 Centre for Research in Occupational Safety and Health, Laurentian University, Sudbury, ON, Canada

**Keywords:** mentors, mentorship program, new licensees, preparedness to practice, quality assurance, veterinarians

## Abstract

Newly graduated and newly licensed veterinarians possess the knowledge and skill required to practice medicine but often struggle to translate that knowledge and skill in a clinical setting without additional supports. The objectives of this exploratory pilot study were to explore the preparedness to practice of newly licensed veterinarians by understanding the perceived potential knowledge or skills gaps and to describe the perceived impact of participation in a peer-facilitated quality assurance program from the perspective of the peer advisors. Semi-structured interviews were conducted with peer advisors (*n* = 10), audio-recorded, transcribed verbatim, and inductively analyzed using Applied Thematic Analysis. Study participants identified perceived gaps in skills and training that may exist for newly licensed veterinarians, including non-clinical professional skills (e.g., communication, self-care) and volume of clinical skills (e.g., species-specific surgery, internal medicine), which may negatively impact their preparedness to practice. Study participants described participation in the peer-facilitated quality assurance program as encouraging self-reflection and continued improvement throughout licensees’ careers rather than traditional in-clinic peer mentorship aimed at improving clinical and non-clinical confidence and proficiency. Study participants described a desire to beneficially influence the profession through helping other veterinarians and their own positive feelings that resulted from their participation in the peer-facilitated quality assurance program. Program participation provided positive experiences for peer advisors and perceived positive experiences for licensees while encouraging learning through self-reflection. There are elements in the Peer Advisory Conversation program that lend itself to being integrated into existing or future peer mentorship programs.

## 1 Introduction

Existing research indicates that veterinarians experience elevated levels of poor mental health outcomes (1), including depression (2), anxiety (3), and burnout (4) compared to the general population (5–7). These mental health challenges are reported to reduce veterinarians’ ability to provide medical care (8) and meet the needs of their clients (9). Challenges have been identified for retention of veterinary professionals in both the USA (10) and UK (11). This attrition from clinical practice is a serious issue because workplace pressures persist, and as more individuals go on to leave or exit the profession, those who remain face an increased risk of poorer mental health, contributing to ongoing cycles of departure.

Several cross-sectional surveys of randomly sampled American Veterinary Medical Association (AVMA) members reported lower levels of well-being among younger veterinarians (12–14). Even with these lower levels of well-being, newly graduated and newly licensed veterinarians possess the knowledge and skill required to practice medicine. However, without additional supports they often struggle to translate that knowledge and skill in a clinical setting (15–17). Newly graduated veterinarians consider mentorship important (18) and a critical aspect to consider during assessment of initial employment opportunities (15). A survey of early career veterinarians in the Netherlands stated 40% believed their mentorship to be moderate, insufficient or poor and overall took 6.8 months for study participants to feel competent to perform five common clinical skills unsupervised (19). With respect to non-clinical skills, some new veterinary graduates reported the most important professional skills for a successful transition into practice were conflict management, client communication, and self-care (18). A survey of veterinarians and veterinary students in Australia reported that some non-clinical professional skills are more important than clinical skills for early career success (20). The increased focus on the importance of these skills is not unique to the veterinary community. A recent scoping review reported that various underrepresented healthcare workers lacked preparedness in non-clinical professional areas which impacted the delivery of healthcare to patients and the well-being of the workers themselves (21).

The College of Veterinarians of Ontario (CVO) regulates the practice of veterinary medicine in Ontario, Canada (22). The College offers a voluntary program called the Peer Advisory Conversation (PAC) where veterinarians are paired with a trained peer advisor (23). The peer advisor is a veterinarian licensed to practice in Ontario, who meets qualification requirements by the CVO to facilitate peer conversations. The training was designed by the CVO to meet the requirements of the PAC program and is not publicly available. The PAC was developed as the core component of the CVO’s quality assurance program to foster public confidence in the profession and provide support to the veterinary profession’s continuing competence. The program focuses on continuing professional development, non-technical skills, medical records review, and case-based discussion. Once a veterinarian is matched with a peer advisor, they submit medical records of four cases to the CVO and complete the Professional Quality of Life Scale (ProQOL) self-assessment tool (24). The completed ProQOL is not submitted to the CVO, but peer advisors will encourage veterinarians to reflect on the results during the PAC. Collection and review of information takes several weeks, and a single meeting is completed in approximately 2 h. The CVO approached our research team to understand whether the PAC initiative could positively impact newly licensed veterinarians (i.e., licensed in Ontario for less than 5 years, including both recent graduates and veterinarians who have recently obtained their license in Ontario). Given the single two-hour meeting the PAC was not developed as a peer-mentorship program (i.e., a relationship where guidance is provided over a period of time) but does share some common elements. The impact of a single-instance peer-advisor (SIPA) program has not been described from the perspective of the peer advisors. Therefore, the objectives of this exploratory pilot study were to understand the perceived preparedness to practice of newly licensed veterinarians and describe the perceived impact of SIPA program participation from the perspective of the peer advisors.

## 2 Materials and methods

### 2.1 Researcher positionality

Researchers must consider how their academic training and social identity influence our understanding and perceptions of the subject under investigation. MB research focuses on the nexus of human well-being and animal welfare, KW research focuses on confidence of new graduates in the veterinary industry, and BG research focuses on occupational and workplace mental health. MB and BG hold a PhD and are experienced qualitative researchers. KW is a practicing veterinarian, and BG is a practicing clinical psychologist. All authors are employed at a veterinary college and have instructed veterinary students: MB as a postdoctoral fellow, KW and BG as assistant professors. The authors maintained an awareness of these positions throughout the research.

### 2.2 Study participant recruitment

The Research Ethics Board at the University of Guelph approved this study (24–07-011). The study findings were reported with the use of the Consolidated Criteria for Reporting Qualitative Research (25). Study participants were recruited by criterion purposive sampling with a recruitment email sent to all those eligible by the CVO (26). Eligible study participants were listed as PAC program peer advisors and licensed to practice veterinary medicine in Ontario, Canada. At the time of the study there were a total of 11 peer advisors active in the PAC program. Demographic information were collected (e.g., age, gender-identity, year in practice, area of veterinary practice, graduate degree) to characterize our sample population.

### 2.3 Data collection

Ten semi-structured interviews were conducted, with nine facilitated by MB and KW and one led by MB alone. Interviews began with a brief introduction of the facilitators which included their background and the research objectives to establish rapport. Study participants were asked questions about challenges faced by newly licensed veterinarians, the effectiveness of the PAC as a potential SIPA program, and the impact that participation in the PAC program had on them. The participants were asked to respond based on their collective experience in the PAC program; however, the number of conversations each participant conducted was not recorded. The semi-structured interview guide was developed by all authors.^1^ Study participants were provided with the guide upon request. Interviews were conducted via and transcribed by Microsoft Teams (version 25122.1201.3625.4656). Subsequently, transcripts were read while listening to recordings by MB to validate accuracy and de-identified. Study participants were provided a $50 gift card upon interview completion.

### 2.4 Data analysis

We approached analysis of this descriptive pilot study through the pragmatist research paradigm since the study focuses on real-world issues and their applied solutions (27). Two interview transcripts were analyzed by MB (NVivo, version 13.7.1, QSR International Pty Ltd.) with applied thematic analysis (28). Codes were developed from the data through iterative analysis, organized into a codebook, and themes were identified. The same two interview transcripts were independently coded by KW (29). These two authors resolved differences in code application through collaborative discussion. The codebook was used by MB to analyze all 10 interviews. The final codebook evolved iteratively throughout analysis (see text footnote 1). Study participants were given anonymous identifiers. Quotations were selected to illustrate a theme or code and words encased in square brackets were inserted for clarity, while ellipsis indicate deleted words.

## 3 Results

### 3.1 Demographic and interview characteristics

A total of 11 veterinarians met our inclusion criteria as active peer advisors. Interviews were conducted with 10 study participants, with three (30%) identifying as men and seven (70%) as women. The average age was 58 years (median = 59, range = 46–68) and average time in veterinary practice was 33 years (median = 33, range = 20–43). Among our study participants, eight (80%) were companion-animal veterinarians, one (10%) was a mixed-animal veterinarian, and one (10%) was a veterinary specialist. Four of our study participants (40%) had a graduate degree (e.g., MSc, PhD, MBA). Interviews were held from January 14 to March 20, 2025. Data collection was stopped once additional insights were unlikely to result from further interviews (i.e., data saturation). Interviews lasted an average of 52 min (median = 52, range = 33–74).

### 3.2 Overall themes

Three themes emerged from the data: (1) perceived gaps in new licensee skills and training, (2) reflections on the PAC as a single-instance peer facilitated program, and (3) impacts on peer advisors themselves.

#### 3.2.1 Perceived gaps in new licensee skills and training

Study participants drew on their professional career experiences as practitioners with newly licensed veterinarians and identified a lack of confidence and skills in both clinical and non-clinical areas of practice. While variation was noted (e.g., *“Everyone has a different set of skills and areas of improvement”* P7), new licensees were described as possessing theoretical knowledge but minimal practical clinical experience, particularly with routine clinical cases and wellness exams (e.g., *“It’s true of any job. You go to school to learn the theoretical stuff and get a little bit of hands-on practical skill. When you get into practice you learn what you really do not know.”* P4). Soft tissue surgery was identified as an area of particular limitation, as explained by this study participant:

*“[Surgery] is a massive gap when they graduate. Particularly after the pandemic, folks are graduating with no significant level of skill in that area… I think surgery is beyond [lack of] confidence. They need the skills. Their lack of confidence is more likely an accurate assessment of the training opportunities that they have had and been able to take advantage of.”* P5.

Non-clinical professional skills were voiced by study participants as areas of low confidence and proficiency for new licensees. Some non-clinical skills of concern identified by study participants were the need for strong boundaries for work-life balance, prioritizing self-care, and the willingness to make mistakes and remain resilient. Communication with other veterinary professionals was identified as an area of low sureness and proficiency (e.g., *“Not feeling confident to speak up…The leadership of their team with [task] delegation. Conflict resolution with fellow [veterinary] colleagues”* P9). Study participants described the challenges new licensees faced communicating with clients, as described by P10:

*“Dealing with clients, they particularly seem to shy away from any form of conflict and you are always dealing with conflict in an exam room…Moving [away] from doctor talk so the layperson can understand. They can still use medical terms, but [they need practice] to get it down to the client level.”* P10.

Numerous groups were identified as being responsible for addressing the areas where it was perceived that newly licensed veterinarians lacked confidence and skills. Study participants explained veterinary colleges had a responsibility to reflect, reassess, and address potential gaps in the teaching curriculum. There were different views regarding the role of the new licensee and their employer in addressing these training gaps. Some study participants believed “the individual and their employers” (P2) were primarily responsible, while others questioned the financial feasibility for *“the individual practice to continue to train and mentor these folks when they come out of school”* (P8). Local, national, or international professional veterinary associations, veterinary regulators, and veterinary colleagues were described as having shared responsibility to address the challenges faced by these veterinarians:

*“That’s a tricky question. A lot of it is yourself. Individuals need to take some responsibility for themselves. Certainly, practice owners have some responsibility to new licensees. The professional [veterinary] organizations should hopefully have resources [to provide]. I do not know, maybe the veterinary regulator. Talking to colleagues I find really helps a lot too. We support each other.”* P3.

#### 3.2.2 Reflections on the PAC as a single-instance peer facilitated program

Study participants described an overarching component of the PAC program was to encourage self-reflection by the new licensees (e.g., *“It is trying to have people identify what they need and then think about how they would get there”* P2). Study participants described licensees seemed to have benefitted from using the program to specifically reflect on career goals and how to achieve them or other challenges (e.g., clinical, non-clinical, work-life balance) and how to resolve them, as this study participant explains: *“It gives them something to aim [for]. A bit of a road map for getting back on track. To know where the goal posts are [and] having the goal posts to aim for”* P1. However, study participants described conversations often exceeded the allotted scheduled meeting (e.g., *“I think it’s comprehensive [and] I always need more than 2 h”* P8). It was also stated the time required for licensees to assemble and submit materials (e.g., *“It’s surprising how long it takes to pull together all the different pieces of the records”* P5) and peer advisors to review materials and produce reports (e.g., *“I’m going to guess [peer advisor]s put 8, 10, 12 h into the 2 h conversation”* P6) could be barriers to program uptake. A period in veterinary practice was recommended to gain a threshold of experiences to benefit from the self-reflective program. Study participants varied in their opinion but stated new licensees would benefit from participation in the program after 1 to 5 years of veterinary practice.

Study participants described how the experiences and language they shared with new licensees fostered a connection based in empathy, compassion, and support. Study participants described the program as safe, confidential, and non-judgmental (e.g., *“Conversations start out a little hesitant and within 5 or 10 min the flow of information has changed completely. They figure out it’s a safe place to say whatever you need to say*” P4). However, misconceptions may exist limiting the uptake of the program (e.g., *“A lot of people think [the conversation] is shared with [regulatory] committees. Are they looking at my medical records? Are they scrutinizing me? Could I get in trouble?”* P3). For the new licensees who do engage with the program, study participants state that the provision of information through dialogue or written reports may improve clinical medical practices or awareness and compliance with regulatory requirements. Study participants also describe that the conversations provide reassurance that licensees meet or exceed professional practice expectations, as explained by P1:

*“Being able to touch in with somebody. Talk to another vet and realize you really know what you are talking about. It’s easy to get too judgy on yourself. Talking to another veterinarian and just having that back and forth. You’re talking from the same knowledge base and the same experience. It’s validating. There’s a point about a year out [from graduation] where your confidence really drops and I think this kind of program could be a bit of a pick me up; a check in; a safety net even”* P1.

#### 3.2.3 Impacts on peer advisors themselves

In this theme, study participants report how they were impacted by the PAC. Study participants described how, as peer advisors, they serve as a source of information for licensees and emphasized that sustained continuing education is needed to stay at the forefront of veterinary medicine. (e.g., *“They’re looking to me for resources…It challenges me to keep looking for resources and [be up-to-date on] current standards.”* P2). The program was not seen as a unidirectional benefit or provision of information. Study participants actively profited from their interactions with licensees and described growth in their capabilities and capacity for advisor-ship (e.g., *“I always learn something from them too.”* P6).

Study participants reflected on their involvement with the PAC and expressed a variety of opinions. Study participants described a sense of personal satisfaction, how their own experiences as veterinary professionals were validated by the conversations with their peers, and how this evoked compassion when similar experiences were discussed. A lack of peer advisor performance appraisal aroused feelings of frustration (e.g., *“There’s never any feedback. A little bit to help. Talk less? Talk more? Be more encouraging?”* P6). Though, discussions regarding mental well-being were recognized as important, they also created apprehension (e.g., *“I think that might be a place where we could spend a bit more time. With a different skill set…We can commiserate but we are not trained counsellors,”* P4). Finally, study participants expressed the desire to be “*in service to the profession and contribute*” (P8) to the broader veterinary community. This professional service not only involved improving perception of the regulator but also assist fellow veterinarians address challenges, as explained by P3:

*“I had no mentor my first year out and it was difficult. I moved to a different practice, and I had a wonderful mentor who I still consider a friend and a father figure years later…I wasn’t supported early on in practice and do not want other people to have that, that negative experience. If I can make it better for somebody else, then why would not I?”* P3.

## 4 Discussion

This exploratory pilot study offers nuanced perspectives from peer advisors regarding participation of newly licensed veterinarians in the PAC. Peer advisors reported opportunities for professional development of licensees through self-reflection and expressed positive impact they received through participation. Taken together our results demonstrated how a SIPA program can build professional community with impact on program participants, peer advisors, and the regulator. New licensees who initially sought opportunities for clinical and non-clinical improvement were provided a clearer understanding of their regulator (i.e., more than investigations of public complaints). Peer advisors who sought to contribute to the veterinary profession volunteered for the PAC program and encouraged self-reflection of program participants to identify and address challenges. Similarly, the PAC program offered the regulator an opportunity to communicate the intent of the PAC program to its membership (i.e., assurance of quality practice through a collegial peer conversation separate and apart from investigations).

The PAC provided a forum for discussion of professional challenges in a non-punitive environment. Nearly 80% of new veterinary graduates reported they made mistakes that resulted in less-than-optimal or adverse outcomes for patients, and many of these veterinarians suffered a substantial emotional toll as a result (30). Veterinary professionals are often elite performers, with high standards and critical of their own mistakes (31). Impostor syndrome is characterized by the underestimation of one’s abilities despite opposing evidence, and fear of being exposed as incompetent and a fraud (32). A survey from the UK, USA, and other countries reported that 68% of veterinarians met the clinical threshold for impostor syndrome, with those in practice less than 5 years at greater odds of experiencing it (33). Early career veterinarians report lower levels of mental well-being (12–14) and the PAC, with its elements of mentorship, allows PAC program participants to discuss challenges, obtain feedback, and receive reassurance from peers who can relate to their experiences outside of the workplace and provides a degree of psychological safety. Feelings of distress were reported when veterinarians did not feel safe in their workplace to discuss adverse medical events (34). PAC program participants received guidance on where to improve from peer-advisors outside of their workplace and without fear of consequences. Recent research reported new veterinary graduates stated a good mentor should readily answer questions and empathize with mentee challenges (18). We encourage additional research to assess the impact of SIPA programs on the mental well-being of newly licensed veterinarians.

Peer advisors perceived self-reflection as an effective method for new licensees to navigate professional challenges and achieve their career goals. However, study participants viewed the PAC as an additional support through the SIPA program, rather than a replacement for traditional mentorship programs that aim to build skill, confidence, and proficiency. Our study participants identified perceived gaps in the training of newly licensed veterinarians, including both the volume of clinical content (e.g., species specific surgery, internal medicine) and non-clinical professional skills (e.g., communication, self-care), which may negatively impact their preparedness to practice. These findings corroborate other research citing challenges in preparedness to practice of recently graduated veterinarians (18, 19), medical doctors (35, 36), nurses (37, 38), and various other healthcare workers (21). No single group was identified as being responsible for addressing these training gaps. A shared informal responsibility exists where veterinary colleges (39), employers (40), professional associations (41, 42), and regulators (17, 23) address aspects of preparedness to practice or challenges faced by newly licensed veterinarians. We call for these groups to coordinate mentorship programs, collaborate to address challenges faced by new licensees (i.e., training gaps), and explore how to effectively integrate SIPA programs into existing mentorship programs.

Our study participants described positive experiences facilitating the PAC. There were feelings of satisfaction and personal validation through a peer-facilitated quality assurance program and providing a service to the veterinary profession. Substantial evidence exists that veterinarians experience elevated levels of poor mental health outcomes, including depression, anxiety, and burnout (1–4). SIPA programs may offer veterinarians opportunities to experience positive affect through interactions with licensees, providing them with the reassurance, and career possibilities during an often-challenging period. Furthermore, success of a SIPA program relies on effective recruitment (e.g., relay benefits of peer advisorship) and retention of peer advisors (e.g., streamline program design to minimize overburdening peer advisors). Our results agree with a recent scoping review where mentor-reported benefits of mentoring in a medical setting included increased skills, professional development, increased knowledge, and personal growth, reflection, satisfaction, and enjoyment (43). A meta-analysis found that mentors were more committed to the organization and satisfied with their jobs (44), highlighting the reciprocal interaction described by our study participants. Future research should investigate the barriers and enablers to SIPA program participation and impacts on peer-advisor and licensees mental health.

### 4.1 Limitations

Interpretation of our findings should be in consideration of our study limitations. There was one potential peer advisor that did not participate in the study. While the perspective of this peer advisor may have contributed to additional variation in the description of experiences it is important to assess if an adequate range has been reached (45). We believe our results are likely valid for interpretation as we reached an adequate level of code saturation with the analysis of the tenth study participant interview yielding information redundancy. The perceptions and experiences of the participants may have been influenced by the training they receive and having this information could help to contextualize the results of this study. A limitation of this study is that the training was designed by the CVO to meet the requirements of the SIPA program and is not available for public review. Additionally, our study participants are from one geographical location, typically older than the new licensees (58 years of age), and a large proportion had advanced degrees (40%). Given the qualitative nature of our study, we caution generalization beyond our study participants. We encourage additional research to understand the perceptions of SIPA programs in different geographical areas and peer advisors of differing demographic characteristics.

## 5 Conclusion

PAC participation provided positive experiences for peer advisors and perceived positive experiences for licensees with opportunities for learning through self-reflection. There are elements in the PAC program that lend itself to being integrated into existing or future peer mentorship programs. However, gaps in training of non-clinical skills and volume of clinical skills need to be addressed to improve the preparedness to practice of newly licensed veterinarians.

## Data Availability

All materials used in the analysis (e.g., data, codebook) are available in Borealis, the Canadian Dataverse Repository at: https://doi.org/10.5683/SP3/GO8UJY.

## Footnotes

1
https://doi.org/10.5683/SP3/GO8UJY
